# Factors Associated With Care Workers' Perceived Difficulty in Collaborating With Nurses on Bowel Care in Dementia Group Homes: A Nationwide Survey in Japan

**DOI:** 10.1002/jgf2.70189

**Published:** 2026-10-14

**Authors:** Yumi Fukuyama, Chie Furushima, Syoko Ito, Akiko Akiyama

**Affiliations:** ^1^ Department of Nursing, Faculty of Nursing St. Mary's College Kurume Fukuoka Japan; ^2^ Department of Nursing, School of Health Sciences at Fukuoka International University of Health and Welfare Okawa Fukuoka Japan; ^3^ Department of Social Work Studies, Faculty of Health Sciences Josai International University Togane Chiba Japan; ^4^ School of Nursing, Graduate School of Nursing Nagoya City University Nagoya Aichi Japan

**Keywords:** bowel management, dementia, home care nurse, interprofessional collaboration, Japan, nursing home

## Abstract

**Background:**

This exploratory study aimed to identify factors associated with care workers' perceived difficulty in collaborating with nurses regarding bowel care in dementia group homes (GHs) in Japan.

**Methods:**

We conducted a nationwide cross‐sectional survey from July to October 2025. Using stratified random sampling, questionnaires were mailed to 2000 of 14,202 GHs listed in national open data. Of 437 responses (21.9%), 423 from care workers were included. Perceived difficulty was rated on a 0–10 numeric rating scale and dichotomized as difficulty (scores 6–10) or no difficulty (scores 0–5). Group comparisons and multivariable binary logistic regression were performed. Missing covariates were multiply imputed. A sensitivity analysis treated perceived difficulty as a continuous outcome.

**Results:**

Of the 417 respondents with valid outcome data, 182 (43.6%) reported difficulty. Four factors were significantly associated with perceived difficulty. Older age (adjusted odds ratio [aOR] = 1.044, 95% confidence interval [CI]: 1.020–1.069), a stronger belief in the importance of abdominal warming (aOR = 1.222, 95% CI: 1.077–1.386), and greater uncertainty about when to report bowel‐related concerns to nurses (aOR = 1.282, 95% CI: 1.175–1.398) were associated with higher odds of perceived difficulty. Longer caregiving experience (aOR = 0.939, 95% CI: 0.906–0.973) was associated with lower odds. Sensitivity analysis produced similar results.

**Conclusions:**

Higher perceived difficulty was associated with older age, shorter caregiving experience, and uncertainty regarding the appropriate timing of consultation with nurses. Clarifying when care workers should seek nursing input may support more effective collaboration in bowel care.

## 1 Introduction

Japan is a super‐aged society, and the number of older adults living with dementia continues to increase [1]. Dementia group homes (GHs) provide community‐based residential care for older adults with dementia in home‐like settings. Under Japan's long‐term care regulations, GHs are not required to employ nurses [2]. Care workers therefore play a central role in monitoring residents' health and identifying changes in their condition [3, 4]. Medical and nursing services are provided through collaboration with clinics, hospitals, home‐visiting nursing agencies, and other healthcare providers [4, 5]. In this setting, care workers must recognize changes in residents' conditions and determine when and how to communicate them to nurses and other healthcare providers.

Bowel care is a clinical area in which timely observation and communication are particularly important. Constipation is common among older adults receiving home care and those living in long‐term care facilities [6, 7]. Poorly managed constipation can lead to fecal impaction and other serious medical complications [8, 9, 10]. Residents with dementia may have difficulty communicating pain or discomfort, and constipation‐related discomfort may be expressed through agitation or other behavioral changes [11, 12]. Care workers must therefore monitor bowel movements, stool characteristics, abdominal symptoms, food and fluid intake, physical activity, medication use, and changes in behavior or general condition [7, 13]. Regular laxative use is also common in nursing homes and has been associated with dementia and impaired communication, further complicating bowel assessment and management [14, 15].

Previous studies on bowel care in older adults have emphasized systematic assessment, non‐pharmacological care, medication management, and continuous monitoring [7, 16, 17].

A randomized controlled trial involving older adults with dementia in long‐term care facilities showed that bowel training and appropriate defecation posture improved bowel outcomes, constipation‐related quality of life, behavioral and psychological symptoms of dementia, and caregiver distress [18]. Qualitative studies have further described constipation management as requiring individualized observation, clinical judgment, and coordination among staff members [13], including collaboration between nurses and care workers in Japanese special nursing homes [19]. However, these studies focused on the effectiveness of specific interventions or the processes of bowel‐care collaboration. Factors associated with care workers' perceived difficulty in collaborating with nurses regarding bowel care in GHs remain unclear.

In the present study, perceived difficulty refers to care workers' subjective evaluation of how difficult it is to collaborate with nurses regarding residents' bowel care. Higher perceived difficulty may reflect barriers to effective information sharing, role clarification, and coordinated decision‐making in bowel care. Interprofessional collaboration (IPC) provides a useful framework for understanding this process. IPC involves information sharing, care coordination, role clarification, and collaborative decision‐making [20, 21]. Previous studies in Japanese long‐term care facilities have reported differences between nurses and care workers in perceptions of their respective roles in providing medical care [22] and difficulties perceived by nurses in collaborating with care workers in end‐of‐life care [23]. Relational coordination further emphasizes shared goals, shared knowledge, mutual respect, and timely, accurate, and problem‐solving communication [24, 25]. Professional accessibility, mutual understanding, and clearly defined roles and responsibilities may also facilitate collaboration [26, 27, 28]. However, it remains unclear which care‐worker and facility characteristics and which bowel‐care‐related perceptions are associated with perceived difficulty in collaborating with nurses in GHs.

Therefore, this exploratory study aimed to identify factors associated with care workers' perceived difficulty in collaborating with nurses regarding bowel care in GHs in Japan.

## 2 Materials and Methods

### 2.1 Sample Selection and Participants

A nationwide cross‐sectional study was conducted in Japan from July to October 2025. As of December 2024, 14,202 GHs were listed in the open data of the Nursing Care Service Information Publication System maintained by the Ministry of Health, Labour and Welfare [29]. Using this database as the sampling frame, 2000 GHs were selected by stratified random sampling according to geographical region and corporation type. Geographical regions were classified into nine blocks: Hokkaido, Tohoku, Kanto, Chubu, Tokai, Kinki, Chugoku, Shikoku, and Kyushu/Okinawa. Corporation types included medical corporations, for‐profit corporations, social welfare corporations, cooperative organizations, non‐profit organizations, and other non‐profit entities. One questionnaire was mailed to each selected GH. Each facility was asked to have the questionnaire completed by one care worker who was familiar with bowel‐care practices and collaboration with nurses regarding bowel care.

### 2.2 Measurements

Data were collected using a structured, self‐administered questionnaire developed for this study. The questionnaire consisted of four sections.

The first section collected information on participant, facility, and work‐related characteristics. Participant characteristics included age, sex, years of caregiving experience, and professional position. Facility and work‐related characteristics included type of corporation, region, current number of residents, nurse employment status at the facility, collaboration with external medical institutions, the number of interprofessional meetings attended in the past year, and the number of end‐of‐life care cases in the past year.

The second section consisted of 17 self‐developed items related to bowel care. These items were developed with reference to previous studies and clinical practice guidelines addressing bowel assessment [30], non‐pharmacological bowel care [31, 32, 33], including abdominal warming [34, 35] and defecation posture [18], and interprofessional collaboration in long‐term care [36]. Each item was rated on an 11‐point numeric rating scale ranging from 0 to 10, with higher scores indicating greater agreement. The items covered knowledge and confidence (3 items), perceptions (7 items), and collaboration (7 items). All 17 items were analyzed separately. Items 15–17 were negatively framed and analyzed using their original scores. Item 15, “I find it difficult to collaborate with nurses regarding bowel care,” was used as the primary outcome (dependent variable).

The third section included the seven‐item Relational Coordination (RC) scale [37, 38]. This section was completed only by respondents who collaborated with external nurses. The referent and item wording were adapted to assess communication and relationships between GH care workers and nurses regarding bowel care. Each item was rated on a 5‐point Likert scale ranging from 1 (strongly disagree) to 5 (strongly agree). The mean of the seven item scores was calculated, with higher scores indicating better relational coordination.

The fourth section included the 17‐item Fujita interprofessional Collaboration Scale (F‐IPC) [39], which assesses interprofessional collaboration behaviors in daily practice. Each item was rated on a 5‐point Likert scale ranging from 1 (strongly disagree) to 5 (strongly agree). The mean of the 17 item scores was calculated, with higher scores indicating stronger interprofessional collaboration behaviors.

Both scales were used because they assess complementary aspects of collaboration: the RC scale assesses the quality of communication and relationships between GH care workers and nurses specifically regarding bowel care, whereas the F‐IPC assesses general interprofessional collaboration behaviors in daily practice.

### 2.3 Statistical Analysis

Descriptive statistics were calculated for all variables. In the absence of an established cutoff for Item 15, participants were divided into two groups based on the scale midpoint: a higher perceived‐difficulty group (scores 6–10) and a lower perceived‐difficulty group (scores 0–5). This binary variable was used as the dependent variable.

Participants with valid responses to Item 15 were included in the univariable analyses. Associations with categorical variables were examined using chi‐square tests, and continuous variables were compared using independent‐samples *t*‐tests. The univariable analyses were used to identify candidate variables for the multivariable analysis rather than for confirmatory hypothesis testing; therefore, no adjustment for multiple comparisons was applied.

Variables associated with perceived difficulty at *p* < 0.20 in the univariable analyses were considered candidates for the multivariable logistic regression model. RC scale responses were available only from respondents who collaborated with external nurses; therefore, the RC scale was excluded from the model. The remaining 11 variables were entered simultaneously. Missing covariate data were handled using multiple imputation with 50 imputed datasets, and pooled estimates were obtained using Rubin's rules. The dependent variable (Item 15) was not imputed. Multicollinearity among the independent variables was assessed using variance inflation factors (VIFs). VIFs ranged from 1.058 to 1.409, indicating no evidence of multicollinearity. Adjusted odds ratios and 95% confidence intervals were calculated.

As a sensitivity analysis, the original 0–10 perceived‐difficulty score was analyzed as a continuous outcome using multiple linear regression with the same covariates as the primary multivariable model among participants with complete data.

All statistical analyses were performed using IBM SPSS Statistics for Windows, version 28.0. A two‐sided *p* value < 0.05 was considered statistically significant.

## 3 Results

### 3.1 Participant and Facility Characteristics

Of the 2000 questionnaires distributed, 437 were returned (response rate, 21.9%). 14 questionnaires completed by nurses were excluded because the study focused on care workers. The final analytic sample comprised 423 care workers (Figure 1; Table 1).

**FIGURE 1:**
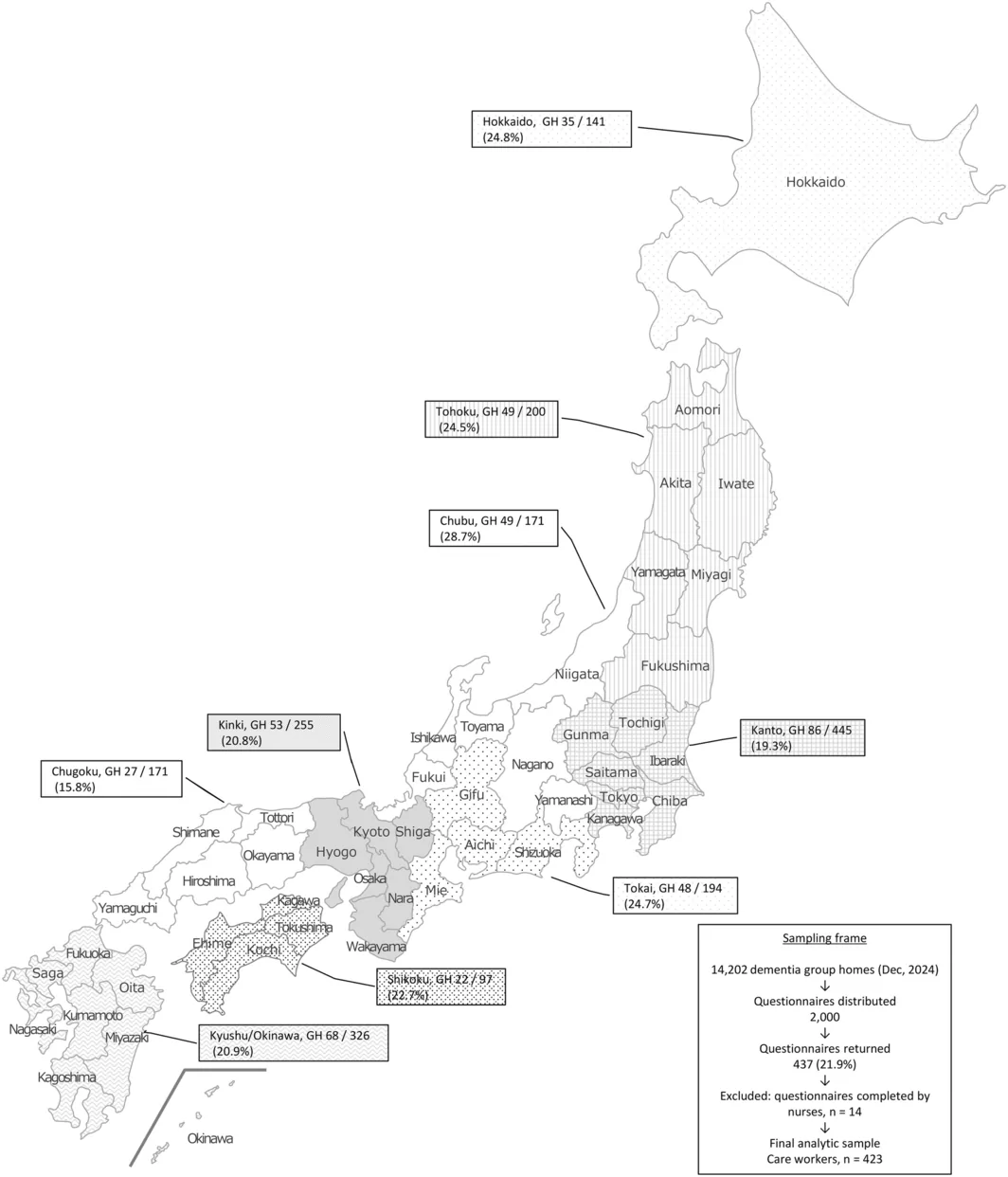
Questionnaire distribution and response rates by region, and participant selection. For each region, values are presented as the number of returned questionnaires/the number distributed and the response rate (%).

**TABLE 1:** Characteristics of participating group‐home staff (*N* = 423).

	*n*	%	Mean ± SD (range)
Demographic and facility characteristics			
Age	413		51.2 ± 10.2 (24–81)
Sex			
Male	163	38.7	
Female	258	61.3	
Years of caregiving experience	419		18.1 ± 6.8 (0–36)
Professional position			
Administrator	283	68.0	
Unit leader	63	15.1	
Staff	46	11.1	
Other	24	24	
Type of corporation			
Medical	67	16.0	
For‐profit	224	53.5	
Non‐profit organization	20	4.8	
Social welfare	100	23.9	
Local government authority	0	0.0	
General incorporated association	0	0.0	
Cooperative association	5	1.2	
Other (religious, educational, or foundation corporation)	3	0.7	
Region			
Hokkaido	35	8.3	
Tohoku	48	11.4	
Kanto	85	20.1	
Chubu	48	11.4	
Tokai	47	11.1	
Kinki	49	11.6	
Chugoku	26	6.2	
Shikoku	22	5.2	
Kyushu/Okinawa	62	14.7	
Current number of residents	418		16.2 ± 6.6 (2–88)
Nurse employment status at the facility			
None	219	54.1	
Part‐time only	116	28.6	
Full‐time only	43	10.6	
Both full‐time and part‐time	27	6.7	
Collaboration with external medical institutions			
Yes	398	94.8	
No	22	5.2	
Care experience and interprofessional activity			
Number of interprofessional meetings attended in the past year	376		1.4 ± 3.4 (0–36)
Number of end‐of‐life care cases in the past year	388		1.6 ± 3.3 (0–50)
Scale scores			
Relational coordination scale (RC)	312		3.7 ± 0.7 (1–5)
Fujita interprofessional collaboration scale (F‐IPC)	408		4.0 ± 0.7 (1–5)

*Note:* Percentages were calculated using available data.

The mean age was 51.2 ± 10.2 years, and the mean caregiving experience was 18.1 ± 6.8 years. Most respondents were administrators (68.0%), followed by unit leaders (15.1%) and staff members (11.1%). Regarding nurse employment at the facilities, 54.1% employed no nurses, 28.6% employed only part‐time nurses, 10.6% employed only full‐time nurses, and 6.7% employed both full‐time and part‐time nurses. Most facilities (94.8%) collaborated with external medical institutions.

### 3.2 Bowel‐Care Knowledge and Confidence, Perceptions, and Collaboration

The highest mean scores were observed for recognizing the importance of observing and recording stool characteristics (Item 6, 9.5 ± 1.1), attention to diet, fluid intake, and physical activity (Item 8, 9.4 ± 1.0), and the impact of bowel care on residents' quality of life (Item 9, 9.4 ± 1.0) (Table 2). Collaboration with nurses was also considered important (Item 11, 9.0 ± 1.5).

**TABLE 2:** Items related to bowel‐care knowledge and confidence, perceptions, and collaboration.

No.	Items	*n*	Mean ± SD (range)
Knowledge and confidence			
1	I understand what to observe during bowel care	423	8.0 ± 1.8 (2–10)
2	I understand how to respond during bowel care	423	7.8 ± 1.8 (1–10)
3	I am confident in making bowel‐care decisions	419	6.5 ± 2.2 (0–10)
Perceptions			
4	I believe abdominal warming is important in bowel care	421	7.4 ± 2.0 (0–10)
5	I believe leaning forward on the toilet is important in bowel care	422	8.1 ± 2.0 (0–10)
6	I believe observing and recording stool characteristics is important	423	9.5 ± 1.1 (1–10)
7	I believe toileting after meals or at scheduled times is important	421	8.5 ± 2.0 (0–10)
8	I believe attention to diet, fluid intake, and physical activity is important	423	9.4 ± 1.0 (4–10)
9	I believe that bowel care greatly affects residents' quality of life	422	9.4 ± 1.0 (5–10)
10	I believe bowel care is an important part of my role	422	8.7 ± 1.6 (3–10)
Collaboration			
11	I believe collaboration with nurses is important in bowel care	422	9.0 ± 1.5 (0–10)
12	I have received advice from nurses about bowel care	414	8.6 ± 2.0 (0–10)
13	I can share my thoughts and observations about bowel care with nurses	418	8.8 ± 1.7 (0–10)
14	I have a trusting relationship with nurses regarding bowel care	414	8.2 ± 2.1 (0–10)
15*	I find it difficult to collaborate with nurses regarding bowel care	417	5.1 ± 3.2 (0–10)
16*	I am unsure about when to report bowel‐related concerns to nurses	412	3.4 ± 2.9 (0–10)
17*	I believe collaboration with nurses is necessary only when a resident cannot defecate	415	2.1 ± 2.6 (0–10)

* Items 15–17 were negatively worded and were not reverse‐coded; original scores are shown. Item 15 was used as the primary outcome and divided into two groups: scores of 0–5 and 6–10.

The mean score for perceived difficulty in collaborating with nurses, the primary outcome, was 5.1 ± 3.2 (Item 15). Among the other negatively worded items, relatively low mean scores were observed for uncertainty about when to report bowel‐related concerns to nurses (Item 16, 3.4 ± 2.9) and the belief that collaboration with nurses was necessary only when a resident could not defecate (Item 17, 2.1 ± 2.6). Among the positively worded items, confidence in making bowel‐care decisions had the lowest mean score (Item 3, 6.5 ± 2.2).

### 3.3 Comparison Between Care Workers With Higher and Lower Perceived Difficulty in Collaborating With Nurses

Of the 423 care workers, 417 provided valid responses to Item 15. Among them, 182 (43.6%) were classified into the higher perceived‐difficulty group and 235 (56.4%) into the lower perceived‐difficulty group (Table 3).

**TABLE 3:** Comparison between care workers with higher and lower perceived difficulty in collaborating with nurses (*n* = 417).

	*n*	Perceived difficulty in collaborating with nurses				*p*‐value
		Higher		Lower		
		*n* = 182 (43.6%)		*n* = 235 (56.4%)		
Sex						
Male	415	64	35.4	96	41.0	0.240
Female		117	64.6	138	59.0	
Age	407	52.5 ± 10.6		50.2 ± 9.9		0.027
Years of caregiving experience	413	17.0 ± 7.0		18.9 ± 6.6		0.006
Participation in interprofessional meetings (times/last year)	370	1.1 ± 2.7		1.6 ± 3.9		0.138
Number of end‐of‐life care cases (times/last year)	382	1.4 ± 2.0		1.9 ± 4.1		0.159
Position						
Administrator	410	123	68.7	154	66.7	0.627
Unit leader		23	12.8	40	17.3	
Staff		22	12.3	24	10.4	
Other		11	6.1	13	5.6	
Type of corporation						
Medical	413	25	14.0	42	17.9	0.270
For‐profit		103	57.5	117	50.0	
Non‐profit organization		6	3.4	14	6.0	
Social welfare		42	23.5	56	23.9	
Cooperative association		3	1.7	2	0.9	
Other		0	0.0	3	1.3	
Current number of residents	412	16.7 ± 7.6		15.8 ± 5.8		0.207
Nurse employment status at the facility						
None	399	102	59.6	112	49	0.043
Part‐time/Full‐time		69	40.4	116	51	
Relational coordination scale (RC)	311	3.7 ± 0.8		3.8 ± 0.7		0.125
Fujita's interprofessional collaboration scale (F‐IPC)	402	3.9 ± 0.7		4.1 ± 0.6		0.043
Knowledge and confidence						
I understand what to observe during bowel care	417	7.9 ± 1.8		8.0 ± 1.8		0.438
I understand how to respond during bowel care	417	7.7 ± 1.9		7.9 ± 1.8		0.162
I am confident in making bowel‐care decisions	413	6.4 ± 2.1		6.6 ± 2.3		0.460
Perceptions						
I believe abdominal warming is important in bowel care	415	7.6 ± 1.8		7.2 ± 2.1		0.020
I believe leaning forward on the toilet is important in bowel care	416	8.3 ± 1.9		8.0 ± 2.1		0.249
I believe observing and recording stool characteristics is important	417	9.4 ± 1.1		9.5 ± 1.2		0.301
I believe toileting after meals or at scheduled times is important	415	8.5 ± 1.8		8.4 ± 2.0		0.536
I believe attention to diet, fluid intake, and physical activity is important	417	9.4 ± 1.0		9.4 ± 1.0		0.654
I believe that bowel care greatly affects residents' quality of life	416	9.4 ± 1.0		9.3 ± 1.1		0.956
I believe bowel care is an important part of my role	417	8.7 ± 1.5		8.7 ± 1.7		0.526
Collaboration						
I believe collaboration with nurses is important in bowel care	417	9.1 ± 1.4		9.0 ± 1.5		0.526
I have received advice from nurses about bowel care	413	8.5 ± 2.1		8.7 ± 2.0		0.284
I can share my thoughts and observations about bowel care with nurses	417	8.7 ± 1.6		8.9 ± 1.7		0.214
I have a trusting relationship with nurses regarding bowel care	414	8.0 ± 1.9		8.3 ± 2.3		0.108
I am unsure about when to report bowel‐related concerns to nurses	412	4.5 ± 3.0		2.6 ± 2.6		< 0.001
I believe collaboration with nurses is necessary only when a resident cannot defecate	415	2.4 ± 2.8		1.8 ± 2.4		0.032

*Note:* *χ* ^2^‐test/*t*‐test, *p* < 0.05.

Compared with care workers in the lower perceived‐difficulty group, those in the higher perceived‐difficulty group were older (*p* = 0.027), had fewer years of caregiving experience (*p* = 0.006), and had lower F‐IPC scores (*p* = 0.043). Nurse employment status at the facility also differed between the groups (*p* = 0.043).

Care workers in the higher perceived‐difficulty group also reported greater recognition of the importance of abdominal warming (*p* = 0.020), greater uncertainty about when to report bowel‐related concerns to nurses (*p* < 0.001), and a stronger belief that collaboration with nurses was necessary only when a resident could not defecate (*p* = 0.032).

### 3.4 Factors Associated With Higher Perceived Difficulty in Collaborating With Nurses

11 variables with *p* < 0.20 in the univariable analyses were entered simultaneously into the multivariable logistic regression model (Table 4). Missing covariate data were handled using multiple imputation, and the analysis included 417 care workers with valid outcome data.

**TABLE 4:** Multivariable logistic regression analysis of factors associated with perceived difficulty in collaborating with nurses regarding bowel care.

Variables	B	*p*‐value	Adjusted OR	95% CI
Age	0.043	< 0.001	1.044	1.020–1.069
Years of caregiving experience	−0.063	< 0.001	0.939	0.906–0.973
Participation in interprofessional meetings (times/last year)	−0.009	0.803	0.991	0.921–1.066
Number of end‐of‐life care cases (times/last year)	−0.050	0.223	0.951	0.877–1.031
Nurse employment status at the facility (none/part‐time or full‐time)	−0.405	0.080	0.667	0.424–1.049
Fujita's interprofessional collaboration scale (F‐IPC)	−0.045	0.815	0.956	0.656–1.394
I understand how to respond during bowel care	−0.011	0.878	0.989	0.862–1.136
I believe abdominal warming is important in bowel care	0.200	0.002	1.222	1.077–1.386
I have a trusting relationship with nurses regarding bowel care	−0.049	0.420	0.952	0.846–1.072
I am unsure about when to report bowel‐related concerns to nurses	0.248	< 0.001	1.282	1.175–1.398
I believe collaboration with nurses is necessary only when a resident cannot defecate	0.017	0.724	1.017	0.927–1.116

*Note:* The multivariable logistic regression analysis included 417 participants with valid outcome data. Missing covariate data were addressed using multiple imputation with 50 imputed datasets, and estimates were pooled using Rubin's rules.

Each additional year of age was associated with higher odds of being in the higher perceived‐difficulty group (adjusted odds ratio [aOR] = 1.044, 95% confidence interval [CI]: 1.020–1.069, *p* < 0.001), whereas each additional year of caregiving experience was associated with lower odds (aOR = 0.939, 95% CI: 0.906–0.973, *p* < 0.001).

A stronger belief in the importance of abdominal warming was associated with higher odds (aOR = 1.222, 95% CI: 1.077–1.386, *p* = 0.002). Greater uncertainty about when to report bowel‐related concerns to nurses was also associated with higher odds (aOR = 1.282, 95% CI: 1.175–1.398, *p* < 0.001).

The other variables included in the model were not significantly associated with higher perceived difficulty.

In a sensitivity analysis treating the original 0–10 perceived‐difficulty score as a continuous outcome, the results were consistent with those of the primary analysis. Older age (B = 0.053, *p* = 0.002), fewer years of caregiving experience (B = −0.067, *p* = 0.006), greater recognition of the importance of abdominal warming (B = 0.270, *p* = 0.002), and greater uncertainty about when to report bowel‐related concerns to nurses (B = 0.484, *p* < 0.001) were associated with higher perceived‐difficulty scores.

## 4 Discussion

In this study, 43.6% of care workers were classified into the higher perceived‐difficulty group. Higher perceived difficulty was associated with older age, shorter caregiving experience, a stronger belief in the importance of abdominal warming, and greater uncertainty about when to report bowel‐related concerns to nurses. Similar results were observed in the sensitivity analysis treating perceived difficulty as a continuous outcome.

Care workers recognized the importance of observing and recording stool characteristics, attending to diet, fluid intake, and physical activity, and collaborating with nurses. Nevertheless, greater uncertainty about when to report bowel‐related concerns to nurses was associated with higher perceived difficulty. A qualitative study in nursing homes described bowel care as a complex process requiring individualized observation, clinical judgment, and coordination among staff members [13]. Previous studies have also identified accessibility, mutual understanding, and established communication relationships as important for interprofessional collaboration [28, 40]. Although the F‐IPC score and uncertainty about when to report bowel‐related concerns were both included in the multivariable model, the VIF values indicated no evidence of multicollinearity. However, uncertainty about when to report bowel‐related concerns may itself represent one aspect of perceived difficulty in collaborating with nurses; therefore, the observed association should be interpreted cautiously. Nevertheless, the finding suggests that clarifying when care workers should consult nurses may be an important issue in bowel‐care collaboration.

Nurse employment status at the facility was not significantly associated with perceived difficulty in the multivariable analysis. Whether a nurse is employed at the facility may not adequately represent care workers' actual access to nursing support. In facilities without nurses, consultation may require additional steps, such as contacting external nurses; however, the present study did not assess communication methods, such as telephone or information and communication technology, response time, or ease of access to nurses. Future studies should examine these aspects of accessibility and communication when evaluating nurse–care worker collaboration in GHs.

Longer caregiving experience was associated with lower perceived difficulty, possibly reflecting greater familiarity with residents' usual bowel patterns and consultation needs. Older age, in contrast, was associated with greater perceived difficulty. Because individuals may enter caregiving work at different stages of life, chronological age does not necessarily correspond to longer caregiving experience. Age and caregiving experience may therefore reflect different aspects of care workers' backgrounds.

Among the perception items addressing specific bowel‐care practices, only greater recognition of the importance of abdominal warming remained associated with higher perceived difficulty in the multivariable model. Abdominal warming was included as one of several non‐pharmacological bowel‐care practices based on previous literature [34, 35]. Why this perception was associated with perceived difficulty is unclear. Because the present study did not assess its actual use, frequency, or effectiveness, this finding should be interpreted cautiously.

Recent Japanese guidelines provide structured approaches to diagnosing and managing chronic constipation [41] and assessing colonic fecal retention in nursing care [30]. These guidelines may provide a basis for shared criteria regarding what care workers should report, when they should report it, and how nurses should respond. Establishing explicit consultation criteria may be particularly relevant because uncertainty about when to report bowel‐related concerns was consistently associated with perceived difficulty in both the primary and sensitivity analyses.

## 5 Implications and Limitations

The findings have practical implications. The assessment diagram in the Japanese nursing‐care guideline could be adapted for joint use by home‐visiting nurses and care workers in GHs. By presenting observable signs and decision points, the adapted diagram could help clarify when care workers should consult nurses and support timely assessment and management. Its effectiveness in GHs should be evaluated in future studies.

This study has several limitations. First, the cross‐sectional design did not allow causal inference. Second, all measures were self‐reported, and the conceptual proximity between uncertainty about when to report bowel‐related concerns and the primary outcome may partly explain their association. In addition, although the bowel‐care items were developed from previous research and expert review, their content validity and test–retest reliability were not formally evaluated.

Third, although facilities were asked to select a care worker familiar with bowel care and nurse collaboration, respondents' direct involvement in daily bowel care and nurse collaboration was not assessed. The response rate was 21.9%, and most respondents were administrators, which may have introduced nonresponse and selection biases and limited the generalizability of the findings to frontline care workers. Fourth, missing covariate data were addressed using multiple imputation; however, the resulting estimates depend on the assumptions underlying the imputation model. Fifth, dichotomizing the 0–10 outcome may have resulted in information loss, and the cutoff has not been validated. However, the sensitivity analysis treating the original score as a continuous outcome yielded findings consistent with the primary analysis. Finally, nurse employment status did not capture nurses' accessibility, communication methods, or the nature and extent of the support they provided.

## 6 Conclusion

Among care workers in GHs, 43.6% were classified into the higher perceived‐difficulty group regarding collaboration with nurses in bowel care. Higher perceived difficulty was associated with older age, shorter caregiving experience, and greater uncertainty regarding the appropriate timing of consultation with nurses. These findings suggest that clarifying when care workers should seek nursing input may support more effective collaboration in bowel care.

## Author Contributions

**Yumi Fukuyama:** conceptualization, methodology, investigation, project administration, funding acquisition, supervision. **Chie Furushima:** writing – review and editing, supervision, methodology. **Syoko Ito:** methodology, writing – review and editing, supervision. **Akiko Akiyama:** methodology, writing – review and editing, supervision, investigation.

## Funding

This work was supported by JSPS KAKENHI Grant Number JP22K11230.

## Ethics Statement

This study was approved by the Ethics Committee of the Faculty of Medicine, Saga University (No. R7‐20). The survey cover letter explained the study purpose, voluntary participation, privacy protection, and data confidentiality. Returning the questionnaire was considered to indicate consent to participate.

## Consent

Not applicable.

## Conflicts of Interest

The authors declare no conflicts of interest.

## Data Availability

The data that support the findings of this study are available on request from the corresponding author. The data are not publicly available due to privacy or ethical restrictions.
